# Reidentification of *Decapterus macarellus* and *D. macrosoma* (Carangidae) reveals inconsistencies with current morphological taxonomy in China

**DOI:** 10.3897/zookeys.995.58092

**Published:** 2020-11-18

**Authors:** Liyan Zhang, Jing Zhang, Puqing Song, Shigang Liu, Pan Liu, Cheng Liu, Longshan Lin, Yuan Li

**Affiliations:** 1 College of Marine Sciences, Shanghai Ocean University, Shanghai 201306, China Shanghai Ocean University Shanghai China; 2 Third Institute of Oceanography, Ministry of Natural Resources, Xiamen 361005, China Ministry of Natural Resources Xiamen China; 3 Fujian Institute of Oceanography, Xiamen 361013, China Fujian Institute of Oceanography Xiamen China; 4 Fisheries College, Jimei University, Xiamen 361021, China Jimei University Xiamen China

**Keywords:** DNA barcoding, genetic diversity, mackerel, morphological characteristics, phylogeny, scad, species identification

## Abstract

*Decapterus macarellus* and *D. macrosoma* are economically important pelagic fish species that are widely distributed in tropical and subtropical seas. The two species are often mistakenly identified due to their morphological similarities as described in the Chinese literature on fish identification. In this study, *D. macarellus* and *D. macrosoma* samples were collected in the Eastern Indian Ocean and the South China Sea and reidentified using morphological and DNA barcoding techniques. The characteristics that distinguish the two species primarily include the scute coverage of the straight portion of the lateral line (the most indicative characteristic for classification), the shape of the predorsal scaled area and its location relative to the middle axis of the eye, and the shapes of the posterior margin of the maxilla and the posterior margin of the operculum. The results revealed a large number of misidentified sequences among the homologous cytochrome oxidase (COI) sequences of the two species in the NCBI database and that the genus *Decapterus* may include cryptic species. In terms of genetic structure, the Sundaland has not blocked genetic exchange between *D. macarellus* populations in the South China Sea and the Eastern Indian Ocean, giving rise to a high level of genetic diversity. In this study, we made corrections to the Chinese classification standards for *D. macarellus* and *D. macrosoma* and the erroneous reference sequences in the NCBI database, thereby providing accurate reference points for the future exploration of cryptic species in the genus *Decapterus*.

## Introduction

Fish species of the genus *Decapterus* in the family Carangidae are pelagic fish widely distributed in tropical and subtropical waters around the world and are generally of high economic value. Fishes of the genus *Decapterus* present one free finlet behind the second dorsal fin and the anal fin and varying degrees of scute coverage along the straight-line portion of the lateral line but no coverage along the curved portion of the lateral line. These characteristics make the fishes easily distinguishable from other species of the family Carangidae (Smith-Vaniz 1999). Currently, the genus *Decapterus* includes 11 species worldwide: *D. akaadsi* Abe, 1958, *D. koheru* (Hector, 1875), *D. kurroides* Bleeker, 1855, *D. macarellus* (Cuvier, 1833), *D. macrosoma* Bleeker, 1851, *D. maruadsi* (Temminck & Schlegel, 1843), *D. muroadsi* (Temminck & Schlegel, 1843), *D. punctatus* (Cuvier, 1829), *D. russelli* (Rüppell, 1830), *D. tabl* Berry, 1968, and *D. smithvanizi* Kimura, Katahira & Kuriiwa, 2013 (Kimura et al. 2013).

*Decapterus macrosoma* (shortfin scad) and *D. macarellus* (mackerel scad) are morphologically similar and thus often confused with each other. In Chinese literatures on fish morphological classification, the morphological descriptions of *D. macrosoma* and *D. macarellus* are largely incorrect (Zhu et al. 1962, 1963, 1979, 1985; Cheng and Zheng 1987; Meng et al. 1995); for example, “*D. macarellus* shows a convex posterior end of maxilla, and the majority of the rear straight-line portion the lateral line is covered with scutes” and “*D. macrosoma* shows a truncate posterior end of maxilla, and scutes cover the rear half of the straight-line portion of the lateral line”. These descriptions contradict those from international studies, particularly those of type specimen morphology (Cuvier and Valenciennes 1833; Bleeker 1851; Nakabo 2013). Thus, in this study, samples of *D. macarellus* and *D. macrosoma* were collected from surveys of the fishery resources in the South China Sea and the Eastern Indian Ocean and were morphologically reidentified.

The mitochondrial cytochrome oxidase (COI) gene fragment varies little within species but significantly between species; this fragment can be amplified via polymerase chain reaction (PCR) using universal primers and standardized experimental procedures and is thus employed for DNA barcoding, which has been widely accepted and utilized (Hebert et al. 2003) for identifying species (Li et al. 2019a; Xu et al. 2019), discovering new species and new records (Li et al. 2018; Chao et al. 2019; Wu et al. 2020), identifying cryptic species (Cheng and Sha 2017; Delrieu-Trottin et al. 2018), identifying ichthyoplankton species (Hubert et al. 2015, Li et al. 2017), and detecting invasive species (Hernández-Triana et al. 2019), among other purposes. Therefore, in this study, we employed DNA barcoding to genetically compare *D. macarellus* and *D. macrosoma* and then aligned the sequences with homologous sequences retrieved from GenBank for further analysis. The barrier formed by the Sundaland has caused the differentiation of various fish species, e.g., *Pampus chinensis* (Euphrasen, 1788) (Li et al. 2019b), between the Indian and Pacific Oceans. The question of whether the geographical barrier formed by the Sundaland has also driven species differentiation in the genus *Decapterus* will be addressed in this study based on the samples collected during surveys of the South China Sea and the Eastern Indian Ocean.

In summary, we aimed to reevaluate *D. macarellus* and *D. macrosoma* by combining morphological analysis with molecular genetics to discern the major diagnostic morphological characteristics and correct DNA barcoding for identification and to provide a timeline for the differentiation of the two species. The findings of this study can provide a scientific reference for the classification of fishes in China and the identification of Carangidae fishes and a theoretical basis for the protection, utilization, development and management of *Decapterus* species germplasm resources.

## Materials and methods

### Sample collection

**Figure 1.**
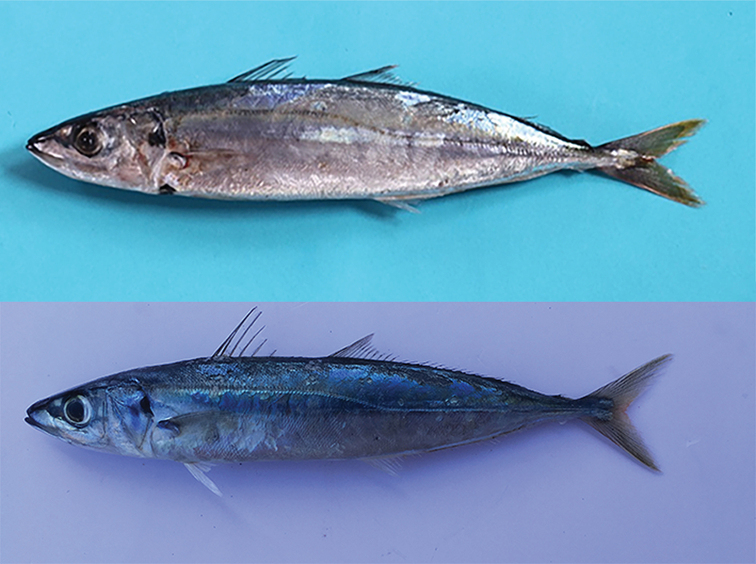
*Decapterus macrosoma* (upper) and *D. macarellus* (lower).

*Decapterus macarellus* and *D. macrosoma* samples were collected from the South China Sea (10°N, 110°30'E) and the Eastern Indian Ocean (2°N, 88°E) in July and October 2019, respectively (Fig. 1); both species were collected from the South China Sea with light purse seining, whereas *D. macarellus* samples were collected from the Eastern Indian Ocean using lightnet lifting. Morphological identification of all samples was conducted with reference to Nakabo (2013) and Yamada et al. (2009). From the samples, 24 individuals of *D. macarellus* (A1~A24) and 21 individuals of *D. macrosoma* (B1~B21) from the South China Sea, in addition to 24 individuals of *D. macarellus* from the Eastern Indian Ocean, were randomly selected; the dorsal muscle was excised from each and preserved in 95% alcohol for use in subsequent molecular genetic analysis.

### Morphological analysis

Using the methods of Kimura and Suzuki (1981) and Xu and Huang (1983), morphological measurements and description of the fish samples were conducted. The countable characteristics included spines and rays in the dorsal fin, rays in the pectoral fin, spines and rays in the pelvic fin, spines and rays in the anal fin, rays in the caudal fin, scutes, and vertebrae (counted from X-ray images), and the measurable characteristics included body length and fork length, which were performed using a Vernier caliper with an accuracy of 0.1 mm. The major morphological diagnostic characteristics included the location on the top of the head reached by the scaled area, the distribution of scutes in the straight-line portion of the lateral line, the morphological characteristics of the scutes, the shape of the posterior margin of the maxilla, and the shape of the posterior margin of the operculum.

### Molecular analysis

Genomic DNA was extracted from specimens of both *Decapterus* species with a Qiagen DNeasy Kit and stored at 4 °C. Using universal primers for the mitochondrial COI gene fragment (F2: 5 '-TCGACTAATCATAAAGATATCGGCAC-3’; R2: 5'-ACTTCAGGGTGACCGAAGAATCAGAA-3') (Ward et al. 2005), the targeted fragment was amplified in a 25 μL PCR system consisting of 17.5 μL of ddH_2_O, 0.15 μL of *Taq* DNA polymerase, 2.5 μL of dNTPs (2 mM), 2 μL of 10 × Taqbuffer (with Mg^2+^), 1 μL each of the forward and reverse primers (2 mM), and 1 μL of the genomic DNA template. The following conditions were applied: 4 min of predenaturation at 94 °C, followed by 28 cycles of 94 °C for 45 sec, 50 °C for 40 sec, and 72 °C for 40 sec, with a final extension at 72 °C for 10 min. A negative control was included to detect DNA contamination. The PCR products (3 μL) were analyzed using 1.5% agarose gel electrophoresis (U = 5 V/cm) and were later submitted to Personal Biotechnology Co., Ltd., for purification and bidirectional sequencing.

To ensure the accuracy of the DNA barcoding for the two *Decapterus* species, we retrieved all homologous COI gene sequences of the two species from GenBank (Table 1) to facilitate subsequent comparative analyses. All the obtained sequences were processed and aligned using DNASTAR software (Madison, WI, USA) to ensure consistency. Using *Decapterus maruadsi* and *Trachurus japonicus* as outgroups, a neighbor-joining (NJ) tree of all the sequences was constructed based on the Kimura two-parameter (K2P) model in MEGA 5.0 software (Tamura et al. 2011), and the genetic distances within and among groups were calculated. All the sequences were searched against the NCBI database using BLAST to validate the accuracy of the sequences of the two *Decapterus* species investigated in this study according to the following criteria: a pairwise sequence similarity ≥ 98% indicated the same species, a pairwise sequence similarity = 92~98% indicated the same genus, and a pairwise sequence similarity = 85~92% indicated the same family (Li et al. 2017).

**Table 1.** Information on haplotype, accession numbers, sequence similarity for the samples and sequences in this study.

	Haplotype	Number	Cited dataset from GenBank				Sequences in this study	
			Accession numbers	Scientific species name	sequence similarity (%)	Corrected species name	ID	Scientific species name
Group 1	Hap_5	63	HQ560948, HQ564377, HQ564442, JF493340, JF493341, JF493342, JF493343, JF493346, JX261016, JX261033, JX261126, JX261170, JX261203, JX261215, JX261216, JX261243, JX261268, JX261269, JX261389, JX261442, JX261499, JX261514, JX261515, JX261519, JX261629, KF841444, KP856776, KP856777, KP856778, KU943769, KU943771, KU943781, KY371382, KY371387, KY371390, KY371391, KY371392, KY371393, KY371394, KY371396, KY371397, KY371398, KY371399, KY371400, KY371401, MH085881, MH638661, MH638663	*D. macrosoma*	100	✓	B1, B2, B4, B5, B6, B7, B9, B10, B13, B14, B16, B17, B18, B19, B20	*D. macrosoma*
	Hap_6	6	JX261160, KY371395, MH638795	*D. macrosoma*	100	✓	B3, B11, B12	*D. macrosoma*
	Hap_7	2	JX260997	*D. macrosoma*	100	✓	B8	*D. macrosoma*
	Hap_8	1			99.8		B15	*D. macrosoma*
	Hap_9	1			99.8		B21	*D. macrosoma*
	Hap_20	1	EU514515	*D. macrosoma*	100	✓		
	Hap_21	1	EU514516	*D. macrosoma*	100	✓		
	Hap_24	1	HQ564441	*D. macrosoma*	100	✓		
	Hap_28	2	JF493344, JF493345	*D. macrosoma*	100	✓		
	Hap_32	1	JX261121	*D. macrosoma*	100	✓		
	Hap_33	4	JX261134, KC970467, KY371388, KY371389	*D. macrosoma*	100	✓		
	Hap_34	1	JX261441	*D. macrosoma*	100	✓		
	Hap_35	1	JX261596	*D. macrosoma*	100	✓		
	Hap_38	2	KP266782	*D. macrosoma*	100	✓	7HYS	*D. macrosoma*
	Hap_41	1	KU943770	*D. macrosoma*	100	✓		
	Hap_44	2	KY371383, KY371385	*D. macrosoma*	100	✓		
	Hap_45	2	KY371384, KY371386	*D. macrosoma*	100	✓		
	Hap_51	1	KY802095	*D. macrosoma*	100	✓		
	Hap_54	1	MF541319	*D. macrosoma*	100	✓		
	Hap_55	1	MF956638	*D. macrosoma*	100	✓		
	Hap_56	1	MF956639	*D. macrosoma*	100	✓		
	Hap_59	1	MH638662	*D. macrosoma*	100	✓		
Group 2	Hap_27	1	JF493339	* Decapterusmacarellus *	94.2	*Decapterus* sp. 2		
Group 3	Hap_63	1	MH980014	* Decapterusmacarellus *	96.4	*Decapterus* sp. 1		
Group 4	Hap_1	54	KM986880, KP266765, KU943796, KU943797, KU943798, KY371373, KY371374, KY371376, KY371377, KY371378, KY371380, KY371381, KY570721, KY570723, KY570729, KY570731, KY570733, MF414832, MF414849, MF414876, MH085883, MH085884, MH638676, MH638686, MH638719, MH638731	*D. macarellus*	100	✓	A15, A16, A17, A18, A19, A23, A24, C1, C5, C6, C7, C8, C9, C13, C17, C20, C21, C23, 1CTYS, A4, A10, A11, A12	*D. macarellus*
Group 4			MH638732, MH638733, MH638755, MH638772, MH638781					
Group 4	Hap_2	1			99.8		A20	*D. macarellus*
	Hap_3	8	KY570726, KY570732, MF414875, MH638794, MN257556	*D. macarellus*	100	✓	A14 A21 C15	*D. macarellus*
	Hap_4	1			99.8		A22	*D. macarellus*
	Hap_10	1			99.8		C2	*D. macarellus*
	Hap_11	1			99.8		C3	*D. macarellus*
	Hap_12	1			99.8		C4	*D. macarellus*
	Hap_13	1			99.8		C10	*D. macarellus*
	Hap_14	2	MF541317	*D. macarellus*	100	✓	C11	*D. macarellus*
	Hap_15	2			99.8		C12 C18	*D. macarellus*
	Hap_16	3	KY371375	*D. macarellus*	100	✓	C14 C22	*D. macarellus*
	Hap_17	1			99.8		C16	*D. macarellus*
	Hap_18	1			99.8		C19	*D. macarellus*
	Hap_19	3	KY570727, MH638687	*D. macarellus*	100	✓	C24	*D. macarellus*
	Hap_23	1	HQ564302	*D. macarellus*	100	✓		
	Hap_25	1	JF493337	*D. macarellus*	100	✓		
	Hap_26	1	JF493338	*D. macarellus*	100	✓		
	Hap_36	1	KF009585	*D. macarellus*	100	✓		
	Hap_42	3	KY371372, MH638698	*D. macarellus*	100	✓	A9	*D. macarellus*
	Hap_43	2	KY371379, MH085882	*D. macarellus*	100	✓		
	Hap_46	1	KY570722	*D. macarellus*	100	✓		
	Hap_47	1	KY570724	*D. macarellus*	100	✓		
	Hap_48	1	KY570725	*D. macarellus*	100	✓		
	Hap_49	1	KY570728	*D. macarellus*	100	✓		
	Hap_50	2	KY570730, MH638739	*D. macarellus*	100	✓		
	Hap_52	2	MF414851, MH638756	*D. macarellus*	100	✓		
	Hap_53	1	MF414877	*D. macarellus*	100	✓		
	Hap_57	1	MH119969	*D. macarellus*	100	✓		
	Hap_58	1	MH119978	*D. macarellus*	100	✓		
	Hap_60	1	MH638714	*D. macarellus*	100	✓		
	Hap_61	1	MH638749	*D. macarellus*	100	✓		
	Hap_62	1	MH638771	*D. macarellus*	100	✓		
	Hap_64	1			99.8		17CTYS	*D. macarellus*
	Hap_65	1			99.8		A1	*D. macarellus*
	Hap_66	1			99.8		A2	*D. macarellus*
	Hap_67	1			99.6		A3	*D. macarellus*
	Hap_68	1			99.8		A5	*D. macarellus*
	Hap_69	1			99.8		A6	*D. macarellus*
	Hap_70	1			99.8		A7	*D. macarellus*
	Hap_71	1			99.8		A8	*D. macarellus*
	Hap_72	1			99.8		A13	*D. macarellus*
Group 5	Hap_40	2	KT326329, MF541318	*D. macrosoma*	100	*D. russelli*		
Group 6	Hap_31	1	JQ681500	*D. macarellus*	100	*D. maruadsi*		
	Hap_37	6	KT718513, KT718514, KT718515, KT718516, KT718519	*D. macarellus*	100	*D. maruadsi*	KP266752	*D. maruadsi*
Group 7	Hap_39	1			100		KP267655	*T. japonicus*
Group 8	Hap_22	1	EU514517	*D. macarellus*	100	*S. crumenophthalmus*		
	Hap_29	2	JQ431681, KJ202148	*D. macarellus*	100	*S. crumenophthalmus*		
	Hap_30	1	JQ431682	*D. macarellus*	100	*S. crumenophthalmus*		

Due to a lack of fossil records for fishes from the genus *Decapterus*, it is impossible to precisely determine the timing of their differentiation. In this study, the divergence time of investigated fishes was estimated based on a nucleotide site divergence rate of 1.2% per million years (Bermingham et al. 1997).

To determine whether the *Decapterus* species from the two sides of the Sundaland have differentiated, we assessed the genetic diversity and genetic structure of *D. macrosoma* and *D. macarellus* based on the acquired COI sequences. Specifically, diversity parameters and unrooted minimum spanning tree (MST) data were analyzed using ARLEQUIN software (Excoffier et al. 2005); the MST was constructed with the MINSPNET algorithm with manual correction.

## Results

### Morphological analysis

Based on the correct classification of *D. macarellus* and *D. macrosoma*, countable and measurable characteristics were determined for 50 individuals from each population (Table 2). The results revealed no significant variation in the countable characteristics between the South China Sea population and the Eastern Indian Ocean population for *D. macarellus*, as follows (populations combined): dorsal fin, VII–VIII, I-30~36, 1 finlet; pectoral fin, 20~24; pelvic fin, I-5~6; anal fin, II, I-26~30, 1 finlet; caudal fin, 16~18; scutes, 24~38; and vertebrae, 23~26. The countable characteristics of *D. macrosoma* were as follows: dorsal fin, VII–VIII, I-31~35, 1 finlet; pectoral fin, 20~23; pelvic fin, I-5~6; anal fin II, I-26~30, 1 finlet; caudal fin, 15~18; scutes, 24~38; and vertebrae, 23~26. A comparison of the countable characteristics between the two species showed that most of the characteristics largely overlapped, making it impossible to distinguish these two species.

**Table 2.** Comparison of countable and measurable characteristics of *D. macarellus* and *D. macrosoma*.

Parameters	*D. macrosoma*	*D. macarellus*	
	South China Sea (*N* = 50)	South China Sea (*N* = 50)	Eastern Indian Ocean (*N* = 50)
**dorsal fin**	VII~VIII, I-31~35+1	VIII, I-30~35+1	VII~VIII, I-30~36+1
**pectoral fin**	20~23	20~23	20~24
**pelvic fin**	I-5~6	I-5~6	I-5~6
**anal fin**	II, I-26~30+1	II, I-26~30+1	II, I-27~30+1
**caudal fin**	15~18	16~18	16~17
**scute**	24~38	25~36	24~38
**vertebrae**	23~26	23~25	24~26
**body weight (g)**	9.8~24.4	7.1~23.9	17.2~27.7
**body length (mm)**	92.1~119.3	20.6~114.3	108.2~127.3
**fork length (mm)**	104.3~128.4	29.3~125.1	114.5~134.6

Combining the findings of previous studies (Zhu et al. 1962, 1985; Meng et al. 1995; Nakabo 2013) with observations of the morphological characteristics of the samples in this study, the major diagnostic characteristics of *D. macarellus* and *D. macrosoma* can be summarized as follows: (1) the straight-line portion of the lateral line of *D. macrosoma*, the majority (approximately 3/4) of which is covered with scutes in the rear end, begins below rays 13~14 of the second dorsal fin, and the scutes show no particular external characteristics; in contrast, the straight-line portion of the lateral line of *D. macarellus*, with the rear half covered with scutes, begins below rays 12~13 of the second dorsal fin, and the highest scute is approximately half the eye diameter; (2) The predorsal scales of *D. macrosoma* do not reach the middle axis of the eye, presenting an “m” shape, whereas the predorsal scaled area of *D. macarellus* reaches or extends past the middle axis of the eye, taking on a “∩” shape; (3) The posterior end of the maxilla of *D. macrosoma* is truncated, and the operculum has a straight posterior margin, whereas the posterior end of the maxilla of *D. macarellus* is convex and round, and the operculum has an oblique posterior margin.

### Molecular analysis

**Figure 2.**
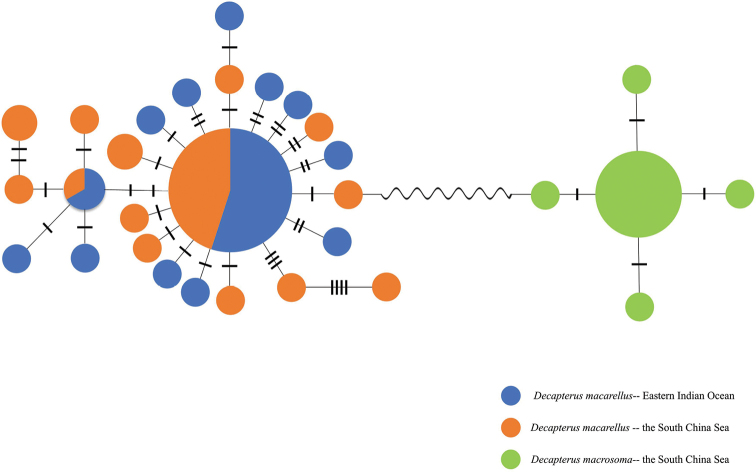
Minimum spanning tree for *D. macarellus* and *D. macrosoma* based on mitochondrial COI sequences.

The 652 bp COI gene fragments from both *D. macarellus* and *D. macrosoma* were amplified using the F2 and R2 primers, and *D. macarellus* exhibits a higher level of genetic diversity than that of *D. macrosoma*. The haplotype diversity (*h*) and the nucleotide diversity (π) were 0.862 ± 0.067 and 0.0037 ± 0.0023, respectively, for *D. macarellus* from the Eastern Indian Ocean; 0.797 ± 0.086 and 0.0030 ± 0.0019, respectively, for *D. macarellus* from the South China Sea; and 0.486 ± 0.124 and 0.0008 ± 0.0007, respectively, for *D. macrosoma* from the South China Sea. The MST constructed based on the COI sequences of the two fish species (Fig. 2) showed that the two species were distinct, with a significant mutation distance. However, the genetic structure did not correspond to the geological locations observed for individuals of *D. macarellus* in the South China Sea and the Eastern Indian Ocean, and there were only two shared haplotypes, one of which was clearly an ancestral haplotype; all other haplotypes were unique to the two seas.

**Figure 3.**
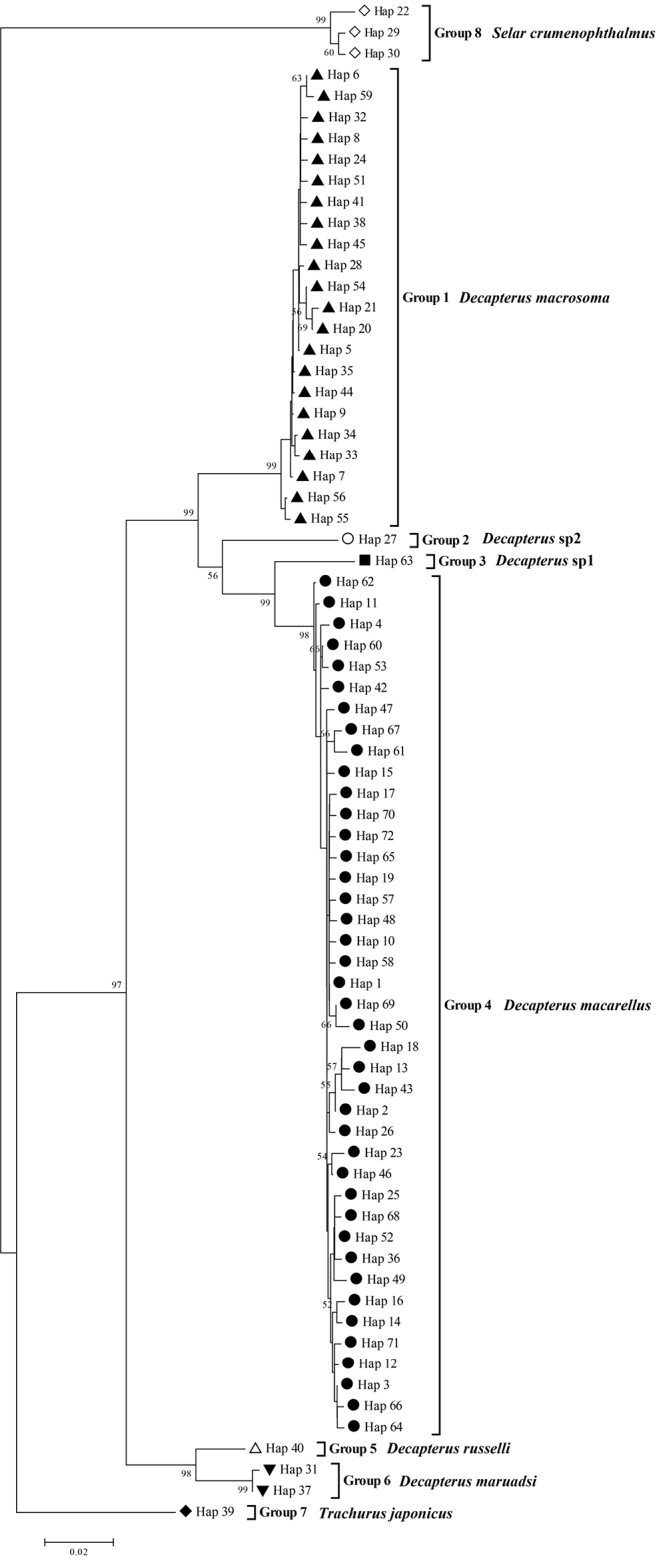
Neighbor-joining tree of detected species based on mitochondrial COI sequences.

After annotating and aligning all the sequences retrieved from GenBank and gained in this study, a 534 bp target fragment was obtained that hosted 142 mutation sites, including 24 single-nucleotide polymorphisms, 118 parsimony-informative sites, and no insertions/deletions. The A+T content was 51.7%, slightly higher than the G+C content, revealing an AT preference. The NJ tree was constructed using all studied sequences with *D. maruadsi* and *T. japonicus* as outgroups (Fig. 3). Eight groups were obtained, with genetic distance among groups ranging from 0.031 (between Groups 5 and 6) to 0.198 (between Groups 3 and 8) (Table 4) and genetic distance within groups of 0–0.009, consistent with the ten-fold rule between species and genera (Ward et al. 2005), which confirmed that each group is a valid species. After realignment, we found that Group 1 corresponded to *D. macrosoma*, Group 2 to *Decapterus* sp. 2, Group 3 to *Decapterus* sp. 1, Group 4 to *D. macarellus*, Group 5 to *D. russelli*, Group 6 to *D. maruadsi*, Group 7 to *T. japonicus*, and Group 8 to *Selar crumenophthalmus*, indicating that the most barcoding of *D. macarellus* and *D. macrosoma* was correct. Notably, for Groups 2 and 3, the highest similarity of the alignment with sequences from the GenBank database was below 95%, which enabled us to assign the species to the genus *Decapterus* but not to identify the species.

Based on a 1.2% nucleotide divergence rate per million years, we estimated the divergence time of the species (Table 4). The results showed that the genetic divergence time of the eight species was in the range of 2.58–16.50 million years, corresponding to the early Miocene Epoch and late Pliocene Epoch. The earliest differentiation appeared between *S. crumenophthalmus* and *Decapterus* sp. 1, and the latest differentiation appeared between *D. russelli* and *D. maruadsi*.

## Discussion

Biodiversity is an important material basis and condition for human survival and sustainable development and usually encompasses species diversity, genetic diversity, ecosystem diversity, and landscape diversity. To study biodiversity, we must first accurately identify the existing species; only with this approach do follow-up studies make sense. For example, both *D. macrosoma* and *D. macarellus* are economically important species in China, but due to historical reasons, the domestic literature on the identification of these two species has been confused, with the species descriptions from China contradictory to those from international literature. In this study, using samples collected in the Eastern Indian Ocean and the South China Sea, we re-examined the two *Decapterus* species from the perspectives of morphology and molecular genetics and provided their major morphological diagnostic characteristics and correct DNA barcoding.

The comparison of countable and measurable characteristics between the two species showed that most of the characteristics are identical or significantly overlapping, making it impossible to distinguish the two species, whereas some directly observable morphological characteristics allow differentiation of the two species (Cuvier and Valenciennes 1833; Bleeker 1851; Nakabo 2013) (Table 3). These characteristics include the scute coverage of the straight-line portion of the lateral line (the most indicative identification characteristic), the shape of predorsal scaled area and its relative location to the middle axis of the eye, and the shapes of the posterior end of the maxilla and the posterior margin of the operculum, among others, indicating that there are appropriate morphological characteristics that enable rapid and correct classification of the two *Decapterus* species. Therefore, correction of the relevant Chinese literature is needed, supporting the significance of the present study.

**Table 3.** Comparison of major morphological diagnostic characteristics of *D. macarellus* and *D. macrosoma*.

	*D. macarellus*	*D. macrosoma*
**straight-line portion of the lateral line covered with scutes**	posterior end, approximately 1/2	majority in the rear, approximately 3/4
**external morphological characteristics of scutes**	the highest scute is approximately half the eye diameter	no particular external characteristics
**whether the predorsal scaled area reaches the middle of the eye**	reaching or extending past	not reaching
**shape of the predorsal scales**	“∩”	“m”
**shape of the posterior end of the maxilla**	convex and round	truncated
**shape of the posterior margin of the operculum**	oblique	straight

The DNA barcoding technique has been repeatedly applied for species identification and has successfully revealed the “cryptic biodiversity” in many taxa (Seidel et al. 2009). In this study, we employed DNA barcoding to reevaluate homologous sequences of *D. macrosoma* and *D. macarellus* and, regrettably, found many errors in the GenBank database. Among the sequences submitted under a scientific name of *D. macrosoma* or *D. macarellus*, we detected seven valid species, including *D. russelli*, *D. maruadsi*, *S. crumenophthalmus*, *D. kurroides*, etc. Moreover, we were unable to identify *Decapterus* sp. 1 and *Decapterus* sp. 2 to species level, since the barcoding sequences of five of the reported 11 species in the genus *Decapterus* have not yet been submitted to the database. Therefore, it is not possible to determine the species level or exclude the possible presence of cryptic species.

**Table 4.** Genetic distance of COI gene among (below the diagonal) and within (on the diagonal) groups, and the divergence time between groups (above the diagonal).

	Group 1	Group 2	Group 3	Group 4	Group 5	Group 6	Group 7	Group 8
***Decapterus macarellus***	0.005	5.92	6.33	5.25	7.17	7.67	10.25	14.75
***Decapterus* sp. 2**	0.071	0	5.67	5.17	8.17	7.42	9.92	15.92
***Decapterus* sp. 1**	0.076	0.068	0	3.00	7.92	7.75	10.08	**16.50**
***Decapterus macrosoma***	0.063	0.062	0.036	0.007	7.50	7.58	11.58	16.00
***Decapterus russelli***	0.086	0.098	0.095	0.09	0	**2.58**	7.75	14.50
***Decapterus maruadsi***	0.092	0.089	0.093	0.091	**0.031**	0.002	8.17	14.67
***Trachurus japonicus***	0.123	0.119	0.121	0.139	0.093	0.098	0	12.33
***Selar crumenophthalmus***	0.177	0.191	**0.198**	0.192	0.174	0.176	0.148	0.009

Unit of divergence time: millions of years.

We estimated the timing of divergence within the genus *Decapterus* to be in the early Miocene Epoch to the late Pliocene Epoch based on the COI nucleotide site divergence rate, which provides a rough timeline for the evolution of species in the family Carangidae. The species in Carangidae originated through differentiation via geographical isolation and adaptive evolution during the diffusion process (Cheng et al. 2011). These two evolutionary processes complemented and interacted with each other, such that the species in *Decapterus* gradually adapted to the surrounding environment and ultimately formed the current geographical distribution pattern.

*Decapterus macarellus* shows significantly higher genetic diversity than *D. macrosoma* and additional mutation characteristics, suggesting that it has higher adaptability, most likely related to its wider distribution. At the level of the COI gene, the genetic differentiation appeared in *P. chinensis* (Li et al. 2019b) was absent in *D. macarellus* from the South China Sea and the Eastern Indian Ocean, indicating that the Sundaland did not block genetic exchange, a result possibly related to the sensitivity of the molecular marker applied in this study and the long-distance migration of the species. We found a large number of unique haplotypes of *D. macarellus* in the two seas, and in the future, we will use more sensitive molecular markers to detect the genetic structure and adaptive evolution of this species in the two seas.

Currently, the shortage of experienced taxonomists capable of completing and updating the descriptions and cataloging work of biodiversity is a major challenge for the scientific community. Species classified by external morphological characteristics are referred to as morphospecies (Primack 2010). It is impossible to correctly classify *D. macrosoma* and *D. macarellus* in China based on morphological characteristics, however, no misidentified sequences corresponding to the morphological classification results were detected among the DNA barcoding data in the NCBI (among which a large number of sequences have been submitted by Chinese investigators from samples collected from various Chinese waters). This is most likely due to DNA barcoding technology maturation and streamlining, which enables investigators to readily obtain targeted sequences that can be aligned with referenced sequences in the database, allowing investigators to overlook the importance of morphology-based classification and instead only refer to data by others.

Initially, species classification primarily depended on the experience of the taxonomist and the accuracy of the literature. However, taxonomists do not necessarily have a background in genetics, whereas geneticists lack expertise in species identification and are unaware of the classification characteristics of the species, resulting in a rift between the two methods. Only by combining the two methods and using DNA barcoding technology as a new identification method enabling the disciplines to complement each other is it possible to classify species rapidly and accurately based on correctly identified morphological characteristics. For example, by combining morphological characteristics and DNA barcoding technology, Li et al. (2019a) accurately classified the *Pampus* species of the world, proposed classification keys for *Pampus* species, and accurately described the distribution of seven *Pampus* species. Using the same strategy, Li et al. (2018) revealed that the originally described *Gymnothorax reticularis* is actually *G. minor*, which is widely distributed in China’s coastal areas, whereas *G. reticularis* is not present in China and is only distributed from the Indian Ocean to the Red Sea. Chen et al. (2018) found that the originally described *Platyrhina tangi* is actually *P. sinensis*, which is present in the coastal area of Zhoushan, China. Therefore, only after correctly identifying a species is it possible to accurately determine the distribution and niche of the species, such that the accuracy of other, related studies can be ensured.

In summary, when identifying fish species, marine biologists need to understand the research status of different taxonomic categories of the fish at home and abroad to ensure the validity of morphological classification. The findings of this study have implications for the classification and evolution of fish species in the genus *Decapterus* and for the conservation of species diversity.

## Conclusion

*Decapterus macarellus* and *D. macrosoma* in the Eastern Indian Ocean and the South China Sea waters were collected and reidentified using morphological and DNA barcoding techniques. The results showed that the morphological diagnostic characteristics of the two species primarily include the scute coverage of the straight portion of the lateral line (the most indicative characteristic for classification), the shape of the predorsal scaled area and its relative location to the middle axis of the eye, and the shapes of the posterior margin of the maxilla and the posterior margin of the operculum. Molecular analysis revealed that both the two species have high genetic diversity, and no genetic differentiation in *D. macarellus* from the South China Sea and the Eastern Indian Ocean was detected. By comparing the COI sequences obtained in this study and those homologous sequences downloaded from GenBank, we speculated that the genus *Decapterus* may include cryptic species and corrected a number of erroneous referenced sequences in the NCBI database.
